# Adjuvanting a subunit SARS-CoV-2 vaccine with clinically relevant adjuvants induces durable protection in mice

**DOI:** 10.1038/s41541-022-00472-2

**Published:** 2022-05-23

**Authors:** Lilit Grigoryan, Audrey Lee, Alexandra C. Walls, Lilin Lai, Benjamin Franco, Prabhu S. Arunachalam, Yupeng Feng, Wei Luo, Abigail Vanderheiden, Katharine Floyd, Samuel Wrenn, Deleah Pettie, Marcos C. Miranda, Elizabeth Kepl, Rashmi Ravichandran, Claire Sydeman, Natalie Brunette, Michael Murphy, Brooke Fiala, Lauren Carter, Robert L. Coffman, David Novack, Harry Kleanthous, Derek T. O’Hagan, Robbert van der Most, Jason S. McLellan, Mehul Suthar, David Veesler, Neil P. King, Bali Pulendran

**Affiliations:** 1 Institute for Immunity, Transplantation and Infection, Stanford University School of Medicine, Stanford University, Stanford, CA USA; 2 Department of Biochemistry and Institute for Protein Design, University of Washington, Seattle, WA USA; 3 Emory Vaccine Center, 954 Gatewood Road, Atlanta, GA 30329 USA; 4 Veterinary Service Center, Department of Comparative Medicine, Stanford, CA USA; 5 Dynavax Technologies Corporation, Emeryville, CA USA; 6 Bill and Melinda Gates Foundation, Seattle, WA 98102 USA; 7 GSK, Rockville, MD USA; 8 GSK, Rixensart, Belgium; 9 Department of Molecular Biosciences, University of Texas, Austin, TX USA; 10 Department of Pathology, Stanford University School of Medicine, Stanford University, Stanford, CA USA; 11 Department of Microbiology & Immunology, Stanford University School of Medicine, Stanford University, Stanford, CA USA; 12 Present Address: Howard Hughes Medical Institute, University of Washington, Seattle, WA 98195 USA

**Keywords:** Vaccines, Immunology

## Abstract

Adjuvants enhance the magnitude and the durability of the immune response to vaccines. However, there is a paucity of comparative studies on the nature of the immune responses stimulated by leading adjuvant candidates. In this study, we compared five clinically relevant adjuvants in mice—alum, AS03 (a squalene-based adjuvant supplemented with α-tocopherol), AS37 (a TLR7 ligand emulsified in alum), CpG1018 (a TLR9 ligand emulsified in alum), O/W 1849101 (a squalene-based adjuvant)—for their capacity to stimulate immune responses when combined with a subunit vaccine under clinical development. We found that all four of the adjuvant candidates surpassed alum with respect to their capacity to induce enhanced and durable antigen-specific antibody responses. The TLR-agonist-based adjuvants CpG1018 (TLR9) and AS37 (TLR7) induced Th1-skewed CD4+ T cell responses, while alum, O/W, and AS03 induced a balanced Th1/Th2 response. Consistent with this, adjuvants induced distinct patterns of early innate responses. Finally, vaccines adjuvanted with AS03, AS37, and CpG1018/alum-induced durable neutralizing-antibody responses and significant protection against the B.1.351 variant 7 months following immunization. These results, together with our recent results from an identical study in non-human primates (NHPs), provide a comparative benchmarking of five clinically relevant vaccine adjuvants for their capacity to stimulate immunity to a subunit vaccine, demonstrating the capacity of adjuvanted SARS-CoV-2 subunit vaccines to provide durable protection against the B.1.351 variant. Furthermore, these results reveal differences between the widely-used C57BL/6 mouse strain and NHP animal models, highlighting the importance of species selection for future vaccine and adjuvant studies.

## Introduction

The rapid development and approval of several COVID-19 vaccines, in less than a year since the emergence of SARS-CoV-2 is unprecedented, and a triumph of modern vaccinology^1^. Sadly, however, there has been a stark gap between the vaccination rates in different counties, with only a small percentage of the population in many developing countries, having received even a single dose of the vaccine^2^. Furthermore, the emergence of several variants of concern, including the B.1.351 lineage first identified in South Africa, has raised concerns that the immune responses induced by the current COVID-19 vaccines may confer only partial or limited immunity against such variants. Therefore, there is an urgent global imperative to develop vaccines with global access, that provide broad protection against variants such as the B.1.351 strain. In this context, vaccine adjuvants hold much promise as they induce enhanced magnitude, durability, and breadth of vaccine-induced immune responses, even with lower doses of antigen^3–13^. As an example, the vaccine candidate NVX-CoV2373, one of the first recombinant protein vaccine candidates with reported human trial results, has highlighted the effect of the adjuvant Matrix M in enhancing vaccine immunogenicity and scaling down the necessary antigen dose^14^. In addition, various adjuvants have been previously shown to increase the durability of humoral responses^12,15–20^.

To date, there are only a handful of clinically approved vaccine adjuvants. Alum, the first adjuvant to have been clinically approved in the 1920s^3^, has been used in multiple vaccines. Since then, few other adjuvants have been approved for use, including AS03—squalene and α-tocopherol containing oil-in-water adjuvant previously used in the influenza pandemic vaccines Pandemrix and Arepanrix^3,21^, CpG1018—a TLR9 agonist used in the Hepatitis B vaccine Hepislav-B^22^. In addition, several other adjuvants have shown promising results in preclinical and clinical evaluations, including AS37—a TLR7 agonist currently in testing for a Meningococcal C vaccine^23^, and Essai O/W 1849101 (hereafter referred to as O/W)—a squalene-in-water emulsion that has been tested preclinically in the context of the RBD-NP vaccine in NHPs. To date, however, the innate and adaptive immune responses stimulated by these different adjuvants, have not been comparatively evaluated. Our recent study evaluated these adjuvants in the context of their capacity to enhance protective immunity to a previously described RBD-NP vaccine against SARS-CoV-2 in non-human primates^3^, but in that study the durability of immune responses induced by the various adjuvants, and whether durable protection against variant strains such as the B.1.351 could be induced by vaccination was not determined. Importantly, in that study only the blood, and not other tissues, was analyzed for immune responses, and analysis of innate responses was not done.

In the current study in mice, we assessed the immunogenicity of the SARS-CoV-2 receptor-binding domain (RBD) nanoparticle vaccine antigen, RBD-I53-50^24^, in combination with five clinically relevant adjuvants (alum, AS03, AS37, CpG1018/alum and as well as a squalene oil-in-water emulsion, Essai O/W 1849101, referred as O/W), in mice. We comparatively evaluated the effect of these adjuvants on humoral and cellular immunity, and the innate immune response induced following immunization with each adjuvant formulation plus antigen. Lastly, we investigated the durability of the neutralizing-antibody responses for 7 months post-immunization and investigated the potential of each adjuvant at enhancing durable protection against infection with a SARS-CoV-2 variant of concern (B.1.351), at 7 months. We find that while all adjuvants enhanced the immunogenicity of RBD-I53–50, each adjuvant formulation induces a distinctive set of innate and adaptive immune responses to the antigen, as well as differences in the durability of humoral immunity and protective immunity to the variant strain of SARS-CoV-2. Importantly our recent results from an identical study in NHPs^3^ allow a benchmarking of clinically relevant adjuvants in mice and NHPs and reveal critical differences in adjuvant performance between C57BL/6 mice and NHPs.

## Results

### RBD-NP immunization with different adjuvants induces robust antibody responses

**Fig. 1:**
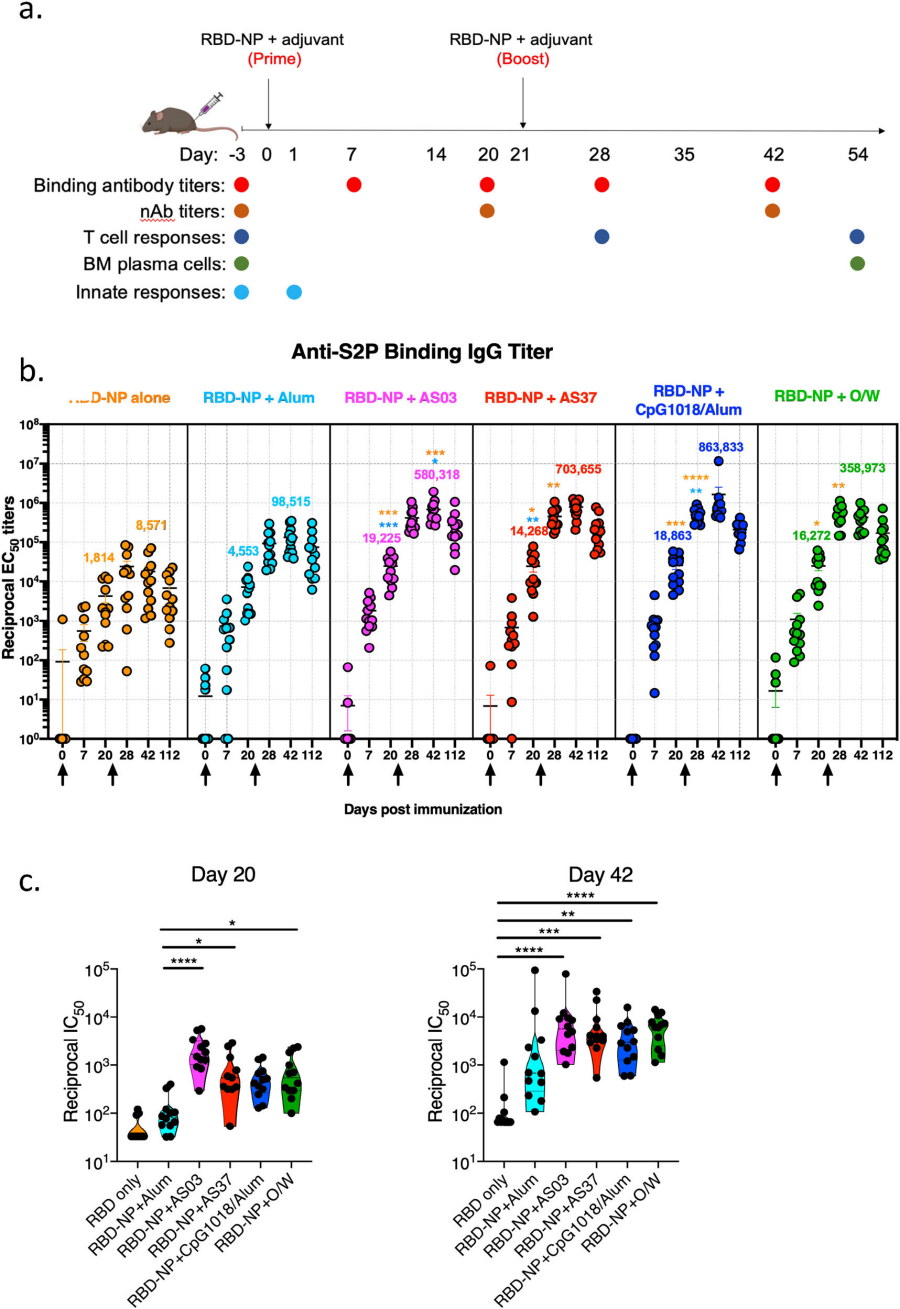
Binding and neutralizing-antibody responses to immunization with RBD-NP and adjuvants. **a** Study design for measuring binding and neutralizing antibodies, T cell responses, BM plasma cells, and innate responses in C57BL/6 mice. **b** Spike-protein binding antibody titers in serum of mice immunized with RBD-NP in various adjuvants at days 0, 7, 20, 28, and 42 post-immunization. Error bars indicate mean ± SEM. **c** Neutralizing-antibody titers measured by pseudovirus neutralization assays at days 20 (prime-only) and 42 (prime-boost). *P*-values: * denotes *p* < 0.0332, **p < 0.0021, ***p < 0.0002, and ****p < 0.0001.

To evaluate the antibody responses to RBD-NP vaccination, we immunized 5 groups of 12 mice each with RBD-NP adjuvanted with alum, AS03, AS37, CpG1018/alum, or O/W at Day 0 and 21. We immunized the sixth group with RBD-NP only (no adjuvant) as a control group. We measured binding antibody responses, pseudovirus neutralizing-antibody responses, as well as the IgG isotypes at day 0, 7, 20, 28, 42, or 112. (Fig. 1a).

The binding antibody responses continued to increase up to day 20 post-primary immunization in all the groups. While the magnitude of the response in the alum-adjuvanted group (GMT of 4553, at day 20) did not differ significantly from that of the non-adjuvanted group, the other four adjuvants induced a 4–5-fold higher magnitude compared to the non-adjuvanted or the alum group (Fig. 1b). The secondary immunization further boosted the response by ~10-fold in all groups, except in the non-adjuvanted group, and peaked at day 42. The magnitude of the response was higher in all four groups compared to the alum or non-adjuvanted groups, and comparable in magnitude to one another (GMTs ranging between 4 and 8 × 10^5^). Importantly, the binding antibody titers persisted up to day 112 post-immunization, with no significant decline in any of the adjuvanted groups.

Consistent with the binding antibody responses, we observed higher neutralizing-antibody responses after a single immunization (day 20) in all the adjuvanted groups, less so in the alum group, than in control non-adjuvanted group (Fig. 1c, left panel). The secondary immunization boosted the response in all the groups. Furthermore, the responses after secondary immunization were significantly higher than in the non-adjuvanted group for AS03, AS37, CpG1018/alum, and O/W. Taken together, our data suggest that all four of the adjuvants induced similarly high binding and neutralizing-antibody titers in mice, with AS03, AS37, and O/W inducing significantly higher early neutralization titers compared to Alum.

### RBD-NP immunization with different adjuvants induces distinct IgG subtypes and bone marrow plasma cell responses

**Fig. 2:**
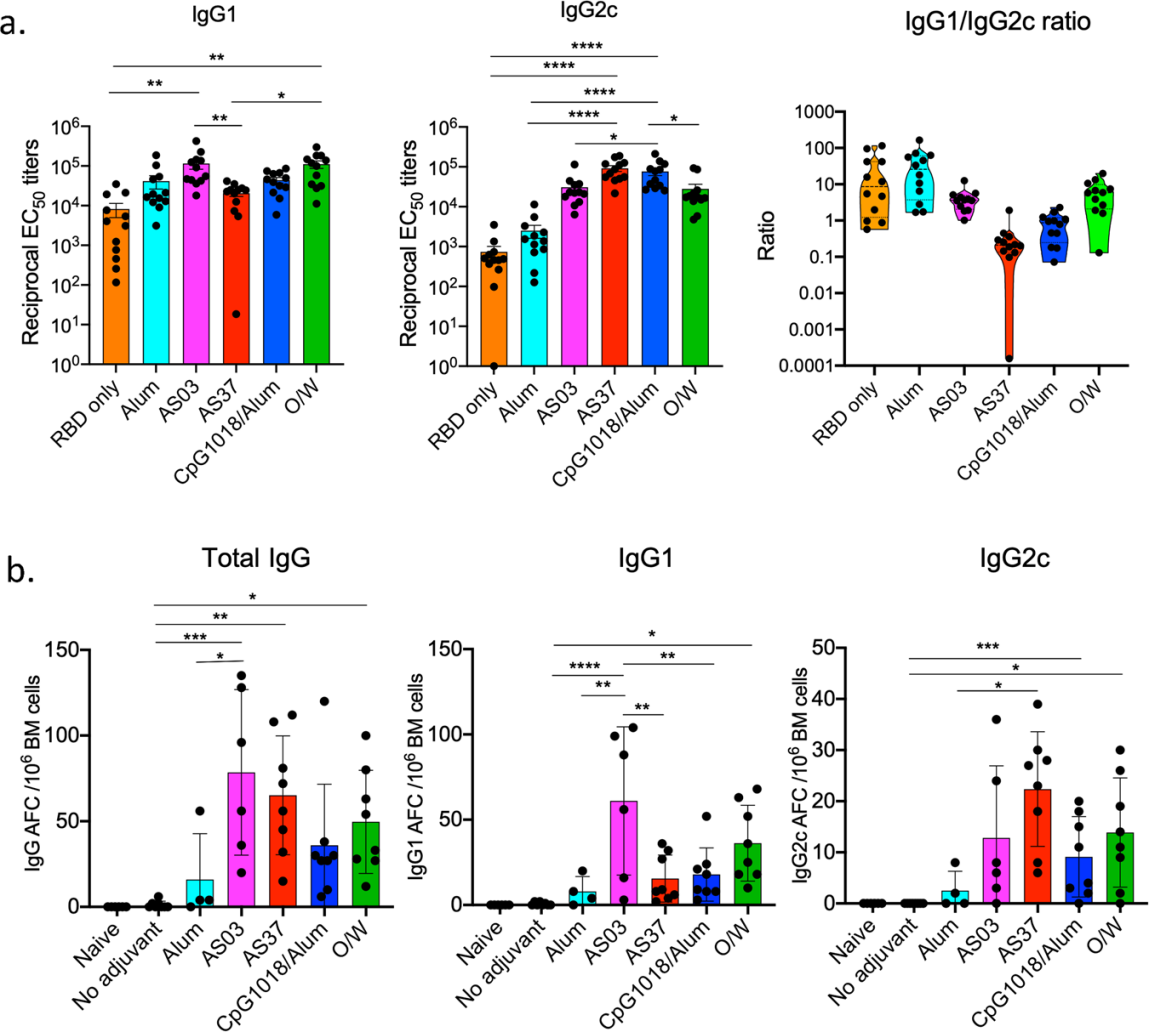
Bone marrow plasma cell responses to immunization with RBD-NP and adjuvants. **a** Anti-spike protein IgG1 and IgG2c titers in serum measured at day 28 post-immunization. Right panel represents ratio of IgG1 to IgG2c titers. **b** Pooled ELISPOT results from 2 independent experiments with *n* = 4 mice per experiment. Total IgG, IgG1, and IgG2c-producing bone marrow plasma cells were quantified at day 33 post-immunization. *P*-values: * denotes *p* < 0.0332, **p < 0.0021, ***p < 0.0002, and ****p < 0.0001. Error bars indicate mean ± SEM in all panels.

In order to investigate the quality of the binding antibody responses induced, we measured IgG subtypes IgG2c and IgG1 at day 28, as a representation of Th1 and Th2-type responses, respectively. Consistent with the literature, alum induced a higher IgG1 response compared to IgG2c^25^. Even though we did not find a difference in the magnitude of the IgG responses between the four adjuvants, strikingly, we did find a different profile of IgG2c versus IgG1 responses induced by these adjuvants. Whereas AS03 and O/W induced significantly higher IgG1 responses compared to AS37 and non-adjuvanted RBD-NP, CpG1018/alum, and AS37 induced significantly higher IgG2c responses, demonstrating a Th1-skewed immune response (Fig. 2a). We further determined the ratio of IgG1/IgG2c responses which demonstrate that while alum induces a Th2-type response, AS03 and O/W promote a mixed Th1/Th2-type response, and AS37 and CpG1018/alum promote a Th1-type response.

In addition, given the durability of the antibody response (Fig. 2b), we measured the frequency of long-lived bone marrow plasma cells which are known to maintain the durability of antibody responses^26,27^. To assess bone marrow plasma cell responses induced by each adjuvant, we immunized five groups of four mice each with RBD-NP adjuvanted with alum, AS03, AS37, CpG1018/alum, and O/W with the same immunization regimen described above (Fig. 1a), with a non-adjuvanted, as well as an unimmunized negative control group. We evaluated the RBD-specific bone marrow plasma cells at day 33 post-boost using an anti-RBD ELISPOT assay. Although no IgG-producing bone marrow plasma cells could be detected in the non-adjuvanted and alum-adjuvanted groups, all four of the novel adjuvant formulations induced bone marrow plasma cells (Fig. 2b), even though in the case of CpG1018/alum, plasma cell numbers were not statistically significantly higher when compared to the non-adjuvanted group. Interestingly, the isotypes of antibodies detected in different adjuvant groups were distinct, with AS37 enhanced Th1-indicative IgG2c plasma cell responses, with little Th2-indicative IgG1 plasma cells, whereas AS03 and O/W induced significant IgG1-, as well as IgG2c-producing plasma cells (Fig. 2b), consistent with the binding antibody response (Fig. 2a).

Thus, our data suggest that despite the high binding and neutralizing-antibody titers induced by all adjuvanted groups, there are qualitative differences between adjuvants in terms of IgG isotypes secreted in the serum, and produced by bone marrow plasma cells, suggesting differences in the Th1 and Th2 biases of the different adjuvants.

### RBD-NP immunization with each adjuvant formulation induces a distinctive pattern of T cell responses

**Fig. 3:**
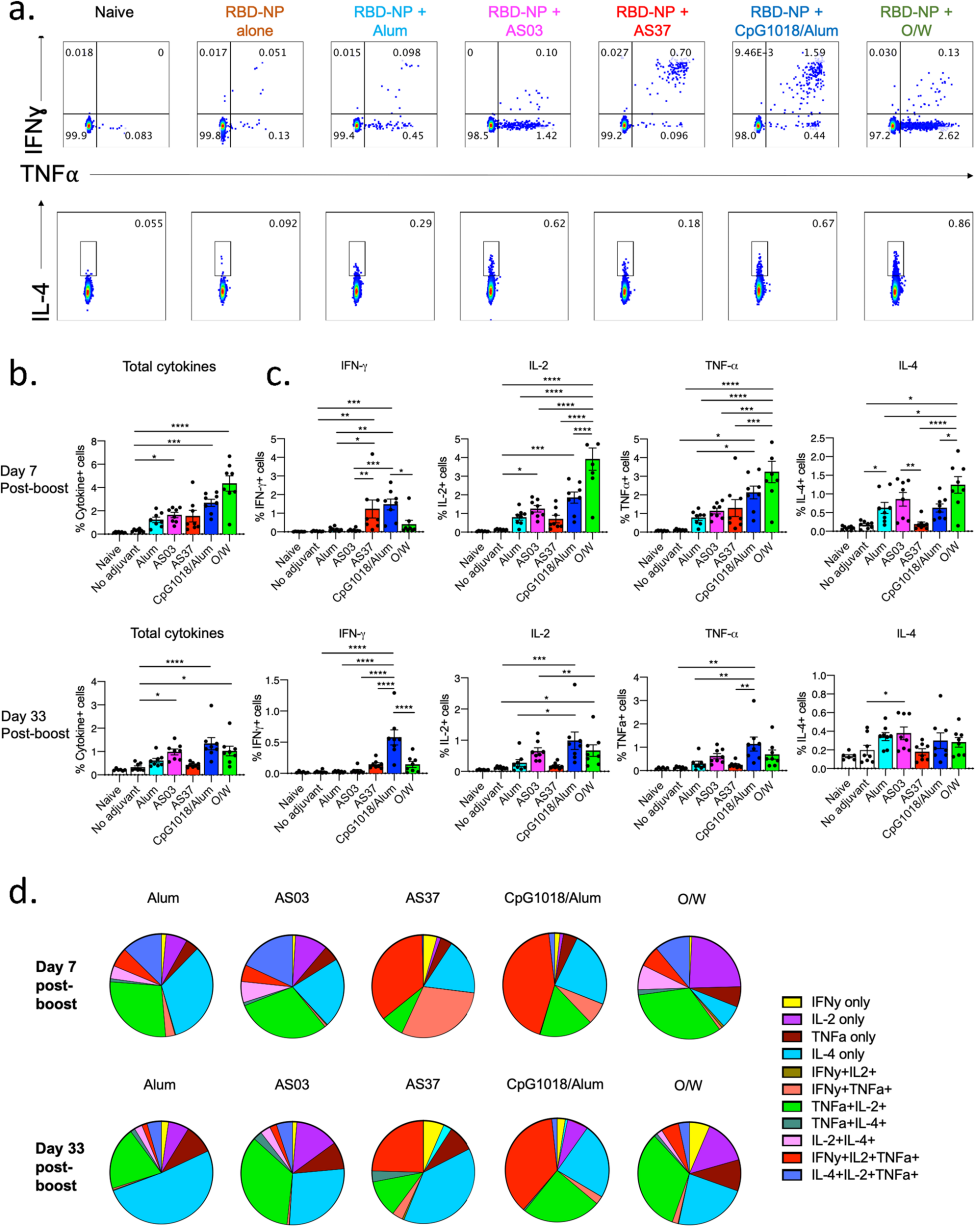
Lung CD4 T cell responses to immunization with RBD-NP and adjuvants. **a** Representative flow cytometry plots of CD4 T cell cytokine production following stimulation with overlapping peptides from SARS-CoV-2 spike protein at day 7 post-immunization. **b** Frequency of total cytokine-producing CD4 T cells at day 7 and day 33 post-immunization. Error bars indicate mean ± SEM. **c** Individual cytokines produced by CD4 T cells in response to overlapping peptide pool at day 7 and day 33 post-immunization. **d** Pie charts representing the average proportions of cytokine-producing cells positive for either one, two or three cytokines. **a–d** Results from 2 independent experiments with *n* = 4 mice per experiment. *P*-values: * denotes *p* < 0.0332, **p < 0.0021, ***p < 0.0002, and ****p < 0.0001.

To evaluate the cellular immune response induced by vaccination, we immunized five groups of four mice each with RBD-NP adjuvanted with alum, AS03, AS37, CpG1018/alum, and O/W with the same immunization regimen described above. A sixth non-adjuvanted group (RBD-NP only) was included, as well as an unimmunized control group. On days 7 and 33 post-boost, lung and iliac draining lymph node cells were isolated and stimulated in vitro with a peptide pool containing overlapping peptides from the SARS-CoV-2 RBD, and IFNγ, TNFα, IL-2, and IL-4 production was detected by intracellular staining following the stimulation (Fig. 3a and Supplementary Fig. 2A). The non-adjuvanted RBD-NP did not induce any detectable cytokine production at either timepoint in lung and LN CD4 T cells (Fig. 3b and Supplementary Fig. 2A). In contrast, all adjuvanted formulations induced significant cytokine production by CD4 T cells at day 7 post-boost, and in all mice, except those adjuvanted with AS37, cytokine production could be detected at day 33 post-boost (Fig. 3b). On day 7 post-boost, IFNγ production by CD4 T cells was detected only in mice that received AS37 and CpG1018/alum (with a mean of ~1.2% and 1.4% IFNγ^+^ cells, respectively, in the lungs) (Fig. 3b and Supplementary Fig. 2A), although no IFNγ could be detected in mice that received AS37 at day 33 post-boost, indicating that AS37-induced cellular immunity may wane earlier when compared to the CpG1018/alum-induced response. In addition, the magnitude of IFNγ response in the group of mice that received CpG1018/alum only decreased ~2-fold in the same time interval, with an average of 0.58% IFNγ^+^ CD4 T cells at day 33 post-boost. In addition, significant TNFα production by CD4 T cells was detected in the groups that received CpG1018/alum and O/W at day 7 post-boost (with ~2.1% and ~3.2% TNFα^+^ CD4 T cells, respectively), however, similar to the IFNγ response, only the group of mice that received CpG1018/alum had detectable TNFα production at day 33 post-boost. Lastly, significant IL-4 production by CD4 T cells was detected in the groups that received O/W and AS03 at day 33, with a similar, although not statistically significant, increase in the AS03 and alum-adjuvanted groups at the earlier timepoint (Fig. 3c).

In addition to differences in individual cytokine production, each adjuvant induced a unique set of multifunctional CD4 T cells. A large proportion of antigen-specific CD4 T cells in mice adjuvanted with alum, AS03, and O/W were TNFα^+^IL-2^+^ double-positive and IL-4^+^ single-positive CD4 T cells, at day 7 post-boost. In contrast, the AS37 and CpG1018/alum adjuvants induced a significant population of IFNγ^+^TNFα^+^IL-2^+^ triple-positive Th1 CD4 T cells at day 7 post-boost. As previously mentioned, the induction of Th1 cells was only durable in CpG1018/alum group, since CD4 T cell responses could not be detected to a significant level in the AS37 group at 33 days post-boost (Fig. 3c). Consistent with our findings, Th1 bias of AS37 and CpG1018/alum adjuvants has been observed in other studies^28–31^, along with less IFNγ production in AS03 and O/W-adjuvanted vaccines^30–33^. With respect to CD8 T cell responses, we observed low levels of cytokine production by CD8 T cells in the alum, CpG1018/alum, and O/W groups, with no significant cytokine production with any of the other adjuvant formulations (Supplementary Fig. 1A, B). We did not observe any IFN-γ production in any of the immunization groups, and although TNF-α production was observed in the alum, CpG1018/alum, and O/W groups on day 7 post-boost, this was no longer detectable at 33 days post-boost (Supplementary Fig. 1C and Supplementary Fig. 2B). Taken together, our data suggest that the five adjuvants tested differ in their ability to induce CD4 and CD8 T cell responses, with AS37 and CpG1018/alum stimulating a Th1-biased response, while AS03 and O/W stimulate a mixed Th1/Th2 response.

### Different adjuvant formulations stimulate distinctive innate immune responses in the draining lymph nodes

**Fig. 4:**
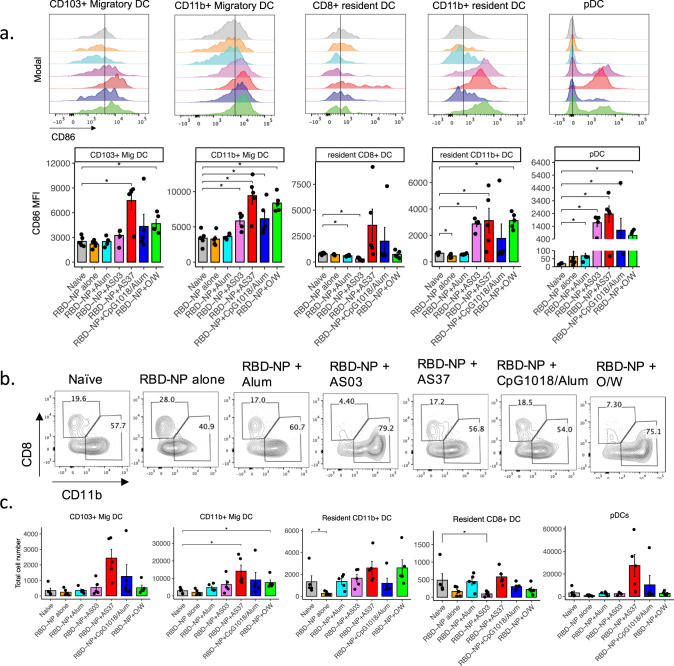
Activation of DC subsets in response to immunization with RBD-NP and adjuvants. **a** CD86 MFI of dendritic cell subsets in the draining iliac lymph nodes 24 h post-immunization. **b** Representative flow cytometry plot for CD8^+^ DCs and CD11b^+^ DCs (gated on resident DCs). **c** Total cell numbers of dendritic cells subsets in the iliac lymph nodes 24 h post-immunization. **a–c** Data representative of 3 in dependent experiments with n = 5 mice per group. *P*-values: * denotes *p* < 0.0332, **p < 0.0021, ***p < 0.0002, and ****p < 0.0001. Error bars indicate mean ± SEM in all panels.

**Fig. 5:**
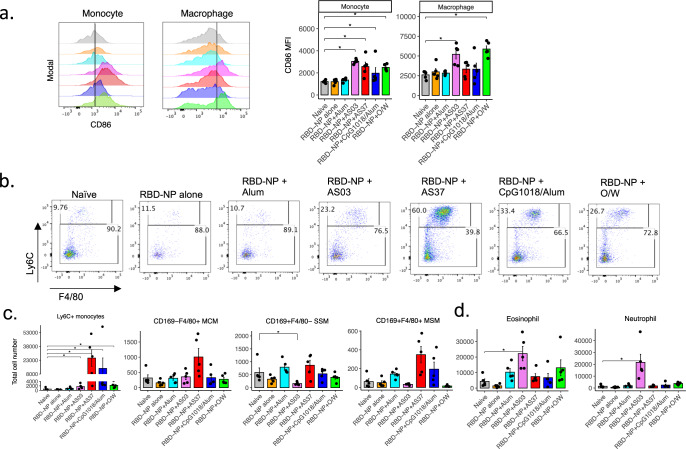
Monocyte, macrophage, and granulocyte responses to immunization with RBD-NP and adjuvants. **a** CD86 MFI of monocytes and macrophages in the draining iliac lymph nodes 24 h post-immunization. **b** Representative flow cytometry plot for Ly6C+ monocytes. **c** Absolute cell numbers of monocytes and macrophage subsets (**c**) and granulocytes (**d**). Data representative of 3 independent experiments with *n* = 5 mice per group. *P*-values: * denotes *p* < 0.0332, **p < 0.0021, ***p < 0.0002, and ****p < 0.0001. Error bars indicate mean ± SEM in all panels.

Our observation that the adjuvants elicit qualitatively distinct humoral and T cell immune profiles pose the question of whether these differences could be mediated by the early innate immune response. Moreover, despite the extensive studies of the effect of these adjuvants on humoral and cellular responses, there is limited understanding of their mechanisms of action, particularly the innate immune responses that are induced. This led us to investigate the activation and recruitment of innate populations in the draining iliac lymph nodes 1-day post-vaccination by flow cytometric staining of various innate populations, including monocytes, dendritic cells, plasmacytoid dendritic cells, neutrophils, eosinophils, and macrophages (Supplementary Fig. 3). Monocytes are identified as CD11b^+^Ly6C^+^ cells, DCs as CD11c^hi^ MHCII^hi^ cells, and DC subsets further subdivided into migratory CD103^+^ or CD11b^+^ DCs and resident CD8α^+^ or CD11b^+^ DCs, LN macrophages as CD11b^+^Ly6C^lo^F4/80^+/−^CD169^+/−^, pDCs as CD11b^−^PDCA-1^+^, neutrophils as CD11b^+^Ly6G^+^ and lastly, eosinophils as CD11b^+^Siglec-F^+^ (Supplementary Fig. 3). To measure the activation of innate cells, subsets of innate cells were evaluated for their upregulation of the activation marker CD86 (Figs. 4a and 5a). We observed that while alum-adjuvanted and non-adjuvanted RBD-NP did not induce any significant activation of classical DCs. The other adjuvants induced a distinct pattern of early innate responses, with enhanced activation of lymph node CD8α + DC by the Th1-biased AS37 and CpG1018 adjuvants, and enhanced activation of lymph node CD11b + DCs by the Th2-biased O/W and SE adjuvants (Fig. 4a). This observation is in line with previous studies that suggest that distinct adjuvants could prime cDC1 or cDC2, which in turn polarizes T cell response towards a Th1 or Th2 response, respectively^34–36^. In addition, we observed significant plasmacytoid DC activation with AS37 and O/W (Fig. 4a). Interestingly, AS03, AS37, and O/W induced significant activation of classical Ly6C^+^ monocytes, and activation of macrophages was also observed with both squalene-based adjuvants, AS03 and O/W (Fig. 5a).

In addition to the activation of innate cells, we sought to investigate the patterns of innate cell recruitment to the draining iliac LNs 24 h following vaccination. In naïve mice, innate cell subsets on average were observed at the following frequencies of total live CD45+ cells in the lymph nodes: monocytes (~0.05%), LN macrophages (~0.08%), migratory DCs (~0.3%), resident DCs (~0.2%), eosinophil (~0.4%), neutrophil (~0.1%). Following immunization, we observed a modest increase in migratory CD11b^+^ DC numbers in the O/W-adjuvanted group (Fig. 4c). Interestingly, LN-resident CD8^+^ DCs significantly decreased in the AS03-immunized group, while an increase in CD103^+^ migratory DCs could be observed in the AS37-immunized group, although not statistically significant (*P* = 0.067) (Fig. 4b). Monocyte recruitment to the draining lymph nodes was also observed at 24 h post-vaccination in AS37, CpG1018/alum, and O/W groups, as measured by the increased frequencies and numbers of Ly6C^+^ monocytes in these groups (Fig. 5b)*.* Notably, AS03 also led to ~4-fold reduction in subcapsular sinus macrophage (SSM) numbers in the iliac LN (Fig. 5b), while this was not found in the TLR-agonist adjuvants, AS37 (TLR7) and CpG1018 (TLR9). Furthermore, AS03 induced a high level of granulocyte migration compared to other adjuvants, as reported in the previous studies^37^. In particular, there was ~5-fold increase in eosinophil and ~14-fold increase in neutrophil numbers (Fig. 5b). This difference between the two squalene-based adjuvants AS03 and O/W could be mediated by the fact that AS03 contains α-tocopherol, which has been shown to be immunostimulatory^37^.

**Fig. 6:**
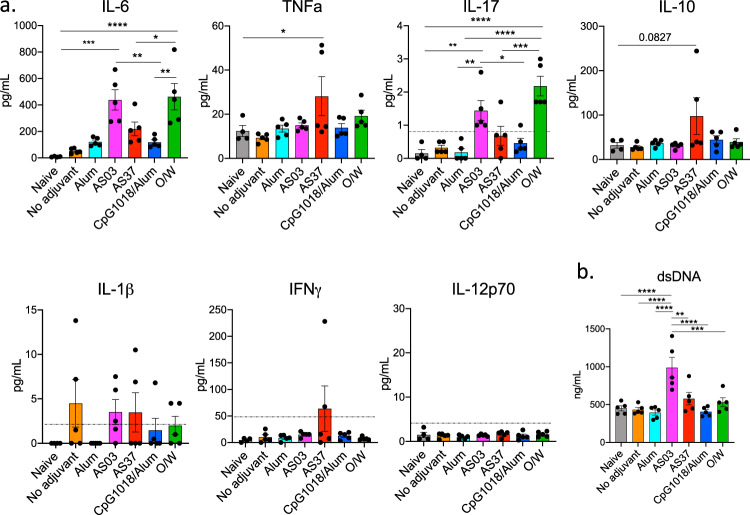
Early cytokine responses following immunization with RBD-NP and adjuvants. **a** Cytokine measurement in serum of mice immunized with each adjuvant formulation at 2 h post-immunization. Serum cytokines analyzed by Simoa multiplex assay. Dashed lines indicate detection limit. If no dashed lines are present, all samples were above detection threshold. **b** Serum dsDNA analysis 24 h post-immunization with each adjuvant formulation. *P*-values: * denotes *p* < 0.0332, **p < 0.0021, ***p < 0.0002, and ****p < 0.0001. Error bars indicate mean ± SEM in all panels.

Lastly, we sought to investigate the early cytokine response to each adjuvant formulation. To this end, we immunized five mice per group with each RBD-NP adjuvant formulation as previously described, and assessed the production of IL-6, together with IL-1β, TNF-α, IL-17, IL-10, and IL-12p70 with a highly sensitive SIMOA multiplex detection assay 2 h post-immunization (Fig. 6a). Whereas no significant IL-1β production was observed, significant IL-6 production could be observed, with the highest levels observed in the sera of mice immunized with the squalene-based adjuvants AS03 and O/W (Fig. 6a). In addition, low levels of early IL-17 production were observed with a similar pattern—the highest IL-17 induction in AS03 and O/W-adjuvanted groups. In contrast, a higher level of TNF-α and IL-10 was observed in AS37 group whereas we did not observe any change in IL-12p70 levels at 2 h post-immunization. Lastly, dsDNA levels in serum were assessed 24 h following immunization as a correlate of cell death. Immunization with AS03 resulted in a significant increase in serum dsDNA, while other adjuvant formulations did not result in a significant increase of dsDNA (Fig. 6b). Overall, our results suggest that different adjuvants induce distinct patterns of innate immune activation, which could potentially mediate the differences observed in the cellular and humoral responses to vaccination.

### Immunization with other clinically relevant antigens highlights the potential of adjuvants in inducing robust antibody responses at lower antigen doses

To evaluate whether similarly robust binding antibody titers can be induced with other clinically relevant SARS-CoV-2 antigens, we compared RBD-NP immunization with different doses of soluble HexaPro^38^, as well as HexaPro displayed on the I53–50 nanoparticle (hereafter referred to HexaPro-NP). The HexaPro antigen is a prefusion-stabilized SARS-CoV-2 spike protein that contains six proline substitutions, making it highly thermostable and suitable for high-yield production while preserving antigenicity^38^. In order to evaluate the antibody responses induced by the different antigens, we immunized mice with the same immunization regimen as above (Day 0 and Day 21), with RBD-NP (1.5 μg), and varying doses of soluble HexaPro and HexaPro-NP (1.5 μg and 0.4 ug for each antigen), adjuvanted with either AS03 or CpG1018/alum. These adjuvants were chosen because both of them are approved for clinical use and induced a high magnitude of antibody responses in mice, and in non-human primates^3^.

At 7 days post-primary immunization, the soluble HexaPro antigen induced significantly lower titers (GMT of 18.4 at 1.5 μg dose) compared to RBD-NP (GMT of 1906.9) in the AS03 immunization group, and HexaPro-NP (GMT of 2917.4 at 1.5 μg dose) in the CpG1018/alum immunization group (Supplementary Fig. 4). At 20 days post-primary immunization, however, soluble HexaPro performed similarly to HexaPro-NP (GMT of 55,691 at 1.5 μg dose), although the RBD-NP antigen outperformed other antigens in the AS03 immunization group (GMT of 139,157). Interestingly, however, this RBD-NP advantage was not observed with antigens adjuvanted with CpG1018/alum (Supplementary Fig. 4).

Following secondary immunization, at day 28, soluble HexaPro induced significantly higher titers (GMT of 1,465,317 at 1.5 ug dose) compared to HexaPro-NP (GMT of 349,436 at 1.5 ug dose) with both AS03 and CpG1018/alum adjuvants (Supplementary Fig. 4). More importantly, at day 42, soluble HexaPro antigen, even at the lower 0.4 ug dose, induced significantly higher binding antibody titers (GMT of 760,492 at 0.4 ug dose) than the 1.5 ug HexaPro-NP (GMT of 233,268 at 0.4 ug dose), and was not significantly different from the 1.5 ug RBD-NP antigen (GMT of 573,792), independently of the adjuvant used (Supplementary Fig. 4). Taken together, our data indicate that even with low doses of antigen, AS03 and CpG1018/alum adjuvant formulations induce robust serum antibody responses against the SARS-CoV-2 spike protein. Moreover, it highlights the potential of the highly thermostable soluble HexaPro as a COVID-19 vaccine antigen capable of producing robust antibody responses when combined with AS03 or CpG1018/alum.

### RBD-NP immunization with different adjuvants induces durable antibody responses and confers protection against a variant of concern

**Fig. 7:**
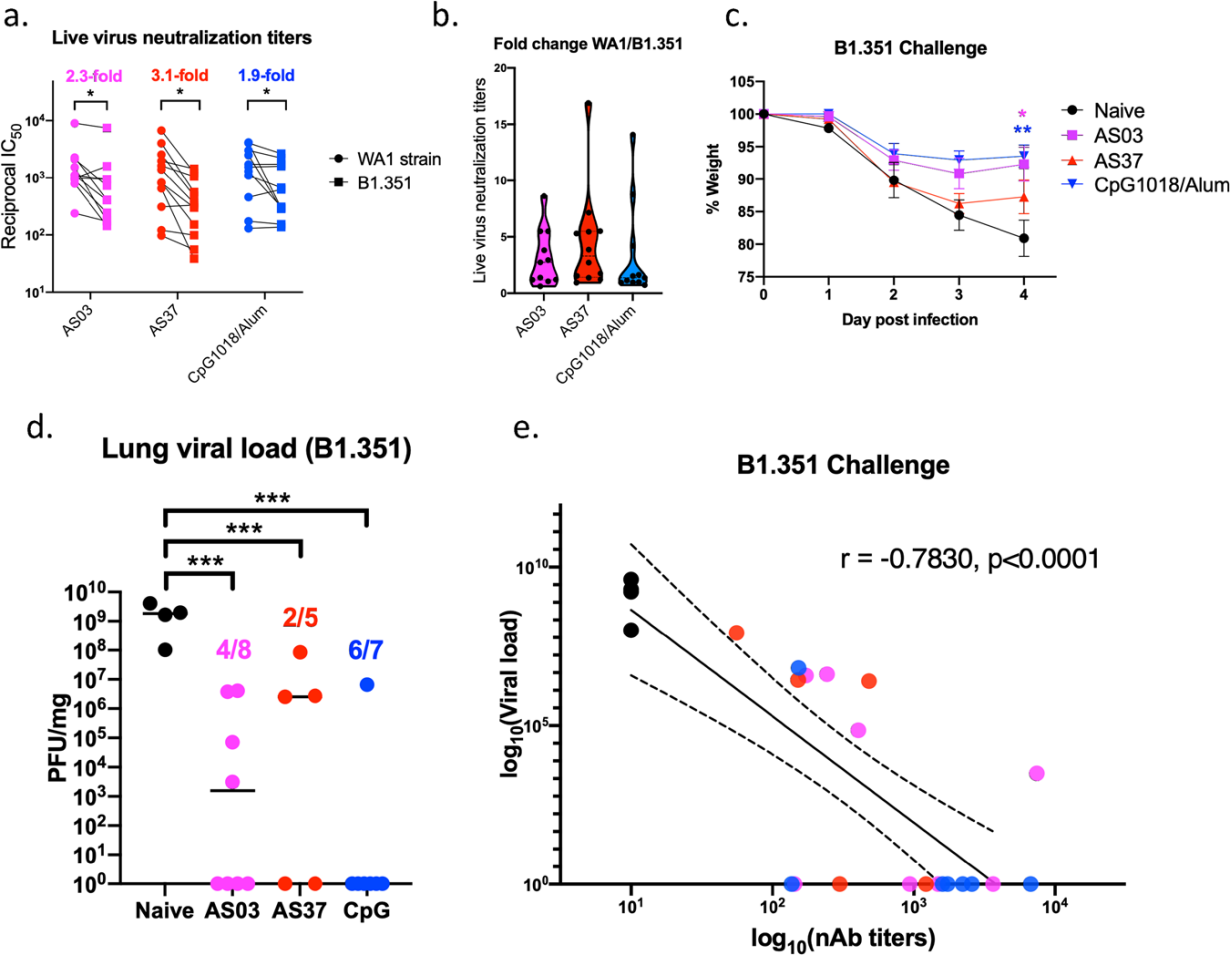
Durability of neutralizing-antibody responses and protection against variants of concern following immunization with RBD-NP and adjuvants. **a** Live-virus neutralizing-antibody titers (GMT, reciprocal IC_50_ values reported) against the WA and RSA strains of SARS-CoV-2 in different adjuvant groups 200 days post-immunization. **b** Fold-change of neutralization titers in different adjuvant groups (WA/RSA). **c** Weight loss following challenge with B.1.351 variant of concern. Error bars indicate mean ± SEM. **d** Lung viral load in all groups following challenge with B.1.351 strain. **e** Correlation of live-virus neutralizing-antibody responses to the B.1.351 strain with viral load (PFU/mg) at Day 4 following challenge with the same strain. *P*-values: * denotes *p* < 0.0332, **p < 0.0021, ***p < 0.0002, and ****p < 0.0001.

To assess the durability of antibody responses following immunization with RBD-NP in different adjuvant formulations, live-virus neutralizing-antibody titers were measured at Day 200 post-immunization (Fig. 7a, b)*.* Neutralization titers were measured against both the original SARS-CoV-2 isolate (WA-1) and the B.1.351 variant. Three adjuvant formulations, namely AS03, AS37, and CpG1018/alum, were used in this experiment. Interestingly, all adjuvant formulations induced durable neutralization titers against both viruses isolates 200 days after vaccination (Fig. 7a). However, the titers induced against the B.1.351 variant were significantly lower in all adjuvant groups. While AS03 and CpG1018/alum groups had an average fold-change of 2.3- and 1.9-fold, respectively, the AS37 group had a higher drop in titers against the VOC (3.1 fold) (Fig. 7a, b). Thus, while all three adjuvant formulations induced durable nAb responses to both the original isolate and a VOC, AS37 had a slight disadvantage for the B.1.351 strain—a result observed in the non-human primate model but to a greater extent^3^. In addition, antibody binding to nine SARS-CoV-2 variants was measured at Day 200 post-immunization, using Mesoscale IgG assay (see Methods). As shown in Supplementary Fig. 5A, all adjuvant formulations induced similar patterns of binding of serum antibodies to the nine variants. Fold changes were calculated by dividing the variant’s binding AUC by that of the WT strain (Supplementary Fig. 5B). Although all adjuvant formulations induced binding antibody responses against RBD from all nine variants, there were some slight differences in the fold changes of certain adjuvants against particular variants. For example, O/W group had a higher decrease in binding to the Beta (K417N, E484K, N501Y), Eta/Iota/Zeta (E484K), and Epsilon (L452R) RBD. However, there was a statistically significant increase in RBD binding from B1.1.7 + E484K strain in the O/W-adjuvanted group (Supplementary Fig. 5B). Moreover, a decreased binding (relative to WT strain) to RBD from Alpha (B.1.1.7, N501Y) strain was observed in the AS37 group. Lastly, a statistically significant difference between the fold changes of CpG1018/Alum and AS03-adjuvanted groups could be observed with regards to binding to the Delta variant of SARS-CoV-2 (L452R, T478K), with CpG1018/Alum outperforming AS03 (Supplementary Fig. 5B).

To investigate protection in vaccinated mice at a late timepoint, C57BL/6 mice immunized with RBD-NP in AS03, AS37, or CpG1018/alum was challenged with the B.1.351 strain of SARS-CoV-2 at Day 200 following immunization. Challenge of the mice with the B.1.351 VOC induced on average 20% weight loss in unvaccinated mice, and significantly lower weight loss in the CpG1018/alum and AS03 groups (Fig. 7c). However, mice receiving the AS37-adjuvanted RBD-NP lost comparable weight to naïve mice, highlighting differences in the adjuvants with respect to their protective capacity against VOCs (Fig. 7c*)*. Furthermore, quantification of lung viral load following the challenge showed significantly lower viral titers in all adjuvanted groups compared to unimmunized mice (Fig. 7d). However, while ~85.7% of the mice in the CpG1018/alum group were protected (6/7 mice), only 50% (4/8) and 40% (2/5) of the mice in the AS03- and AS37-immunized groups were protected from B.1.351 challenge (Fig. 7d). Lastly, a moderate correlation was observed between the live-virus nAb titers and the viral loads on Day 4 post-challenge (Fig. 7e). Altogether, this data points to differences in the durability and breath of protection against a VOC of SARS-CoV-2, highlighting the importance of adjuvant selection in vaccine design.

### Adjuvant formulations induce distinct responses in mice and non-human primates

In a parallel study, non-human primates were immunized with the exact same antigen-adjuvant combinations following the same timeline (Arunachalam et al.^3^). Some adjuvant formulations induced similar responses in both animal models while others exhibited stark differences between mice and NHPs (Table 1). Firstly, with respect to binding antibody responses, all 4 adjuvants outperformed alum in mice, whereas in NHPs, alum induced comparable responses to AS37 and O/W, while AS03 and CpG1018/alum induced significantly higher binding antibody titers compared to O/W^3^. Secondly, with respect to neutralizing-antibody responses, while O/W induced high nAb titers in mice, it induced lower nAb titers in NHPs relative to the other adjuvants (Fig. 1b, c and ref. ^3^). As for the breadth of antibody responses, AS37 induced a greater fold-change in the neutralization titers between the WA-1 and B.1.351 isolates in both mice and NHPs (Fig. 7a and ref. ^3^). Moreover, a stark contrast in the early induction of neutralizing antibodies was observed in mice compared to NHPs. In mice, a single immunization induced sufficient neutralizing-antibody responses, while the post-prime neutralization titers in NHPs were low (Fig. 1c and ref. ^3^).

**Table 1:** Summary of the adjuvant-induced immune responses observed in this mouse study and a parallel NHP study by Arunachalam et al.^3^.

Parameter	C57BL/6 mice	Non-human primates
Anti-Spike binding antibody titers	GMT at Day 42 for adjuvants range from 350,000 to 800,000, compared to ~100,000 for alum.	GMT at Day 42 ranged from 5000 to 10,000 for all adjuvants except O/W (range of 500–5000).
Peak neutralizing Ab titers (original SARS-CoV-2 strain)	GMT at Day 42 for pseudovirus neutralization titers ranged from 2800 to 8400 for adjuvants, compared to ~500 for alum.	GMT for Day 42 live-virus neutralization assays ranged from 2000 to 4,000 in alum, AS03, CpG1018/alum and AS37 groups, compared to GMT of 244 for O/W.
nAb responses against VOC	2–3-fold drop in GMT nAb titers at Day 200 between WT and RSA strains with AS03, AS37, and CpG1018/alum. (Other groups not assays).	5–8-fold drop in GMT nAb titers between WT and B.1.351 strain with AS03, CpG1018/alum, and alum groups, compared to a 15-fold drop in AS37 group.
Antigen-specific CD4 T cell responses	T cell responses detected in all adjuvant groups.	T cell responses detected in all adjuvant groups except AS37.
Th1 responses	Th1 cytokines detected with AS37 and CpG1018/alum; however, AS37-induced response was not durable.	Th1 cytokines detected with AS03 and CpG1018/alum.
Th2 responses	Th2 cytokines detected with AS03, O/W, and alum.	Th2 cytokines detected with AS03 (both Th1 and Th2 responses observed with AS03).
Protection against challenge	(Only VOC B.1.3.5.1 challenge performed) Protection observed in all 3 adjuvants tested, with the following hierarchy: CpG1018/alum & AS03 > AS37 > naive mice.	Protection observed in all adjuvant groups, and to a lesser extent in O/W-immunized groups.

Thirdly, CpG1018/alum, O/W, and alum groups had similar patterns of cellular responses in both mice and NHPs, while AS03 and AS37 induced significantly different responses between the two animal models. In NHPs, AS03 induced both Th1 and Th2 cytokines in CD4 T cells, whereas no IFNγ was detected in mouse T cells following in vitro stimulation (Fig. 3c, d and ref. ^3^). In addition, while AS37 induced significant production of Th1 cytokines in mice, no cytokine production could be observed by T cells in NHPs immunized with RBD-NP + AS37. These observations suggest that while C57BL/6 mice are a useful model to assess the immunogenicity of vaccine adjuvants, it is critical to investigate adjuvant immunogenicity in NHPs and humans.

## Discussion

Adjuvants have been shown to enhance the magnitude and durability of vaccine-induced immune responses. However, few studies to date have comparatively evaluated clinical adjuvant candidates, in terms of their potential to achieve the desired immunological outcomes required for protection against the specific pathogen of interest. Moreover, there is a lack of understanding of the mechanisms of action of different adjuvants and how the innate immune response induced by each adjuvant translates into humoral and cellular immune features post-vaccination.

In the case of a novel pathogen like SARS-CoV-2, the correlates of protection against infection are yet to be formally established. However, several studies of vaccine candidates, as well as COVID-19 patients, have suggested the potential correlates of protection needed. In several vaccine studies, neutralizing antibodies correlated with lower viral load post-challenge in non-human primates^3,39,40^. Regarding the isotype of binding antibodies, in a mouse model of influenza vaccination, it has been shown that both IgG1 and IgG2a/c isotypes are of importance when it comes to protection against challenge^41^. Thus, high magnitudes of anti-spike/RBD binding and neutralizing antibodies are the desired outcome for vaccinations, and induction of a diverse set of IgG isotypes may be potentially beneficial. Furthermore, we and others showed that high titers of neutralizing antibodies against the ancestral SARS-CoV-2 (derived from 3 immunizations of NHPs or vaccination of convalescent individuals) correlate with increased resilience to antigenic drift^42,43^. As for cellular immune responses, Th1-polarized CD4 and CD8 T cell responses are beneficial in fighting viral infection^1^. In a recent study of COVID-19 patients with varying disease severity, it was observed that while nAb titers did not correlate with decreased disease severity, antigen-specific CD4 and CD8 T cell responses did^44^. More importantly, the study highlighted that having more than one arm of the adaptive immune response correlated with less severe disease. Altogether, given this evidence, the ideal adjuvant should induce a high magnitude of humoral immune responses, paired with a coordinated cellular immune response—antigen-specific CD4 and CD8 T cell responses with a Th1 profile, and these responses should be durable in order to achieve protection at late time points post-vaccination.

In this study, we evaluated the immune response to the SARS-CoV-2 RBD-I530-50 nanoparticle vaccine candidate, in combination with the adjuvants alum, AS03, AS37, CpG1018/alum, and O/W. Several studies have shown enhanced antibody production in response to the adjuvants being tested here^23,28–31,45,46^, although a direct comparison between the different formulations had not been performed. When comparing the binding and neutralizing-antibody titers, all four of the adjuvants induced higher titers of antibodies compared to the traditional alum adjuvant, which is consistent with a recent study reporting similar neutralizing-antibody induction induced by AS03 and CpG1018/alum in mice^30^. The isotypes of binding antibodies induced differed with different adjuvants, with AS03 inducing both IgG1 and IgG2c isotypes, while CpG1018/alum and AS37 inducing a higher IgG2c/IgG1 ratio, indicative of a more Th1 response. In fact, a Th1/IgG2a/b bias with AS37 has been previously reported^28^, as well as an IgG1-dominated response in O/W-immunized mice^31^, confirming our findings. Consistent with this, we observed CD4 T cell IFN-γ production only with AS37 and CpG1018/alum. However, TNF-ɑ and IL-2 production in CD4 T cells was still observed with AS03 and O/W groups, although both groups also had induction of IL-4 in CD4 T cells. Thus, all adjuvants induced early CD4 T cell responses, with CpG1018/alum and AS37 showing the most Th1 profile. It is important to note, however, that late T cell responses (33 days post-boost) were only observed with the CpG1018/alum immunization, which has implications for the durability of these responses. Altogether, all adjuvants induced comparable magnitude of serum antibody responses (both binding and neutralizing), but a distinct set of cytokines in the cellular immune compartment, with CpG1018/alum having the most durable Th1 response.

To understand whether the adaptive differences observed in the T cell compartment could be reflective of differences in the innate immune response by the different adjuvants, we measured the activation of innate cells in all groups. In particular, AS37, one of the adjuvants that appeared to induce a more Th1-prone response as measured by IFNy+TNFa+IL2 + CD4 T cell frequencies as well as IgG2c isotype of binding antibodies, also appeared to mount a higher cDC1/cDC2 ratio in the lymph nodes at 24 h post-immunization. This is consistent with the notion that cDC1s induce a more Th1 response, and that activation of multiple DC subsets results in the induction of a higher diversity of IgG isotypes. In addition, although AS03 and O/W are both squalene-based adjuvants, immunization with AS03 resulted in a significant reduction of SSM macrophages, while O/W did not induce a similar effect on the SSM. Interestingly, other squalene-based adjuvants have previously shown loss of macrophage subsets^47,48^, and each squalene-based adjuvant has been shown to affect different macrophage subsets differently, thus, this difference observed between AS03 and O/W is not unexpected and can be attributed to the differences in their formulation. Together, our data provide some preliminary understanding of the innate mechanisms engaged by each adjuvant; however, the exact innate pathways induced, as well as how they direct the adaptive immune responses, remains a question of interest and should be further elucidated. In addition, how our results would translate into the human setting remains unclear, due to the differences in pattern recognition receptor expression between mice and humans. For example, TLR9, the receptor targeted by CpG1018, is more widely expressed in mouse innate cell types, whereas in NHPs and humans, TLR9 is expressed only in pDCs and B cells^49^. Interestingly, as summarized in Table 1, our parallel adjuvant studies in mice and NHPs point to clear differences between the immune responses observed in the two animal models. Thus, evaluation of each adjuvant in non-human primates and humans is warranted and will provide additional insight into the use of these adjuvants in human vaccines.

Lastly, since the durability of immune responses is vital to developing successful vaccines, we assessed neutralizing-antibody titers 200 days following immunization in the AS03, AS37, and CpG1018/alum groups, and observed significant nAb responses both against the original SARS-CoV-2 isolate and a VOC (B.1.351). Since SARS-CoV-2 variants have been of great concern, mice were challenged at a late (7 month) timepoint with the B.1.351 variant. While the CpG1018/alum and AS03 adjuvants induced significant protection against the B.1.351 variant, no significant reduction in weight loss was observed with AS37 adjuvant, although AS37 immunization resulted in a significant reduction in lung viral load. These observations are in line with the results in the non-human primate study of these adjuvants^3^. This observation was in line with the higher fold-change in nAb titers between the original and RSA strains of SARS-CoV-2 with AS37, compared to other adjuvants, since a correlation could be observed between the nAb titers and viral load following the challenge. While nAb titers correlated significantly with protection in this study, the extent to which the Th1 responses observed solely in the CpG1018/alum group contribute to the enhanced broader protection remains to be investigated, as it is difficult to draw conclusions about the contribution of cellular immune responses in the presence of high neutralizing-antibody responses, which was the case in our study. It is noteworthy to mention that the B.1.351 challenge studies were performed with C57BL/6 mice, and while the mice experienced weight loss and significant viral replication in the lungs the following challenge, the overall pathogenesis of SARS-CoV-2 is likely different from that in permissible animal models such as NHPs. Nevertheless, the protective capacity of the adjuvanted subunit vaccine at a 6-month timepoint against this variant of concern is encouraging. Overall, this study shows some of the key differences in the immunity induced by various clinically relevant adjuvant formulations, highlighting the importance of selecting the most efficacious adjuvant in vaccine design.

## Methods

### Antigen-adjuvant formulations and immunizations

The RBD-NP, soluble HexaPro, and HexaPro-NP immunogens were produced as described^3^. For immunizations with RBD-NP antigen, for each mouse, RBD-NP was diluted to 60 ug/mL (RBD component) in 25 uL to achieve a final amount of 1.5 ug antigen per mouse (~4.3 ug total mass of RBD-I53–50 including the scaffold). The Vaccine Formulation Institute (VFI) established the formulation of RBD-NP with O/W. The dose of O/W was 50% v/v, and the adjuvant was mixed with RBD-NP (60 ug/mL) diluted with Phosphate buffer saline with 150 mM NaCl.

AS03 and AS37 adjuvants were kindly provided by GSK Vaccines. AS03 is an oil-in0water emulsion containing 11.86 mg α-tocopherol, 10.69 mg sqyualene, and 4.86 mg polysorbate 80 (Tween-80) in PBS. AS37 is a TLR7 agonist (200 ug/mL) adsorbed to Aluminium hydroxide (2 mg/mL). For the AS03 and AS37 formulations, RBD-NP was diluted to 60 mg/mL in 25 uL tris-buffered saline (TBS, 50 mM Tris, pH = 8, 150 mM NaCl) and mixed with an equal volume of AS03 and AS37.

Alum (Alhydrogel 2%) was purchased from Croda Healthcare (Batch #0001610348). Fifty micrograms Alum was used per mouse. For the Alum formulations, 1.5 ug antigen was diluted in TBS and combined with 50 ug Alum and incubated on ice for 30 min.

CpG1018 was generously provided by Dynavax Technologies at a concentration of 12 mg/mL. For each CpG1018/alum formulation, 1.5 ug antigen was diluted in TBS and mixed with 50 ug Alum and incubated on ice for 30 min. After incubation, 10 ug CpG1018 was added to the mix.

Each mouse was immunized in the right hind limb calf (gastrocnemius) muscle with a 50 uL final volume.

### Mice

Eight to fourteen-week-old female C57BL/6 mice purchased from Jackson Laboratories were used for all of the experiments, including the challenge with the B.1.351 variant.

### ELISAs for measuring binding antibody titers

Clear Flat-Bottom Immuno Nonsterile 384-Well Plates (Thermo Scientific, cat. no. 464718) were coated with S-2P antigen (80 ng per well). Plates were blocked with TBS containing 2% BSA for 1 h at 37 °C, then washed and 8–15 dilutions of serum samples were added (depending on the samples being analyzed). Plates were incubated at 37 °C for 1 h, washed 6× with TBS, and 0.05% Tween-20. Secondary HRP-tagged anti-mouse IgG, IgG1, and IgG2c (Southern Biotech) were added at a 1:5000 dilution and incubated for 1 h at 37 °C. Plates were then washed, and KPL SeraCare TMB substrate was added, followed by KPL Stop solution. Absorbance values on plates were read at 405 nm wavelength. Reciprocal EC_50_ titers were calculated by GraphPad Prism.

### Cell lines

HEK293T/17 is a female human embryonic kidney cell line (ATCC). The HEK-ACE2 adherent cell line was obtained through BEI Resources, NIAID, NIH: Human Embryonic Kidney Cells (HEK293T) Expressing Human Angiotensin-Converting Enzyme 2, HEK293T-hACE2 Cell Line, NR-52511^50^. All adherent cells were cultured at 37 °C with 8% CO_2_ in flasks with DMEM + 10% FBS (Hyclone) + 1% penicillin-streptomycin. Cell lines were not tested for mycoplasma contamination nor authenticated.

### Pseudovirus production

Adapted from Walls et al. 2020^24^. MLV-based SARS-CoV-2 S pseudotypes were prepared as previously described^51–53^, except that the SARS-CoV-2 S construct contained the D614G mutation and a truncation of the C-terminal 21 residues^50,54^. Briefly, HEK293T cells were co-transfected using Lipofectamine 2000 (Life Technologies) with an S-encoding plasmid, an MLV Gag-Pol packaging construct, and the MLV transfer vector encoding a luciferase reporter according to the manufacturer’s instructions. Cells were washed 3× with Opti-MEM and incubated for 5 h at 37 °C with a transfection medium. DMEM containing 10% FBS was added and incubated for 60 h. The supernatants were harvested by a 3000 × *g* spin, filtered through a 0.45 μm filter, concentrated with a 100 kDa membrane for 10 min at 3000 × *g,* and then aliquoted and stored at −80 °C. A detailed protocol for pseudovirus production can be found at Millet and Whittaker, 2019^51^.

### Pseudovirus entry and Serum Neutralization assays

Adapted from Walls et al. 2020^24^. HEK-hACE2 cells were cultured in DMEM with 10% FBS (Hyclone) and 1% PenStrep with 8% CO_2_ in a 37 °C incubator (ThermoFisher). One day prior to infection, 40 μL of poly-lysine (Sigma) was placed into 96-well plates and incubated with rotation for 5 min. Poly-lysine was removed, plates were dried for 5 min then washed 1× with water prior to plating cells. The following day, cells were checked to be at 80% confluence. In a half-area 96-well plate a 1:3 serial dilution of sera was made in DMEM in 22 μL final volume. 22 μL of pseudovirus was then added to the serial dilution and incubated at room temperature for 30–60 min at room temperature. HEK-hACE2 plate media was removed and 40 μL of the sera/virus mixture was added to the cells and incubated for 2 h at 37 °C with 8% CO_2_. Following incubation, 40 μL 20% FBS, and 2% PenStrep containing DMEM was added to the cells for 48 h. Following the 48–72 h infection, One-Glo-EX (Promega) was added to the cells in half culturing volume (40 μL added) and incubated in the dark for 5 min prior to reading on a Varioskan LUX plate reader (ThermoFisher). Measurements were done on all sera samples from each group in at least duplicates. Relative luciferase units were plotted and normalized in Prism (GraphPad) using a zero value of cells alone and a 100% value of 1:2 virus alone. Nonlinear regression of log(inhibitor) vs. normalized response was used to determine IC_50_ values from curve fits.

### Cells and viruses

VeroE6-TMPRSS2 and VeroE6-TMPRSS2-hACE2 cells were kindly provided by Dr. Barney Graham (Vaccine Research Center, NIH, Bethesda, MD). The generation of TMPRSS2-hACE2 cells with lentivirus encoding hACE2-P2A-TMPRSS2 was previously described^55,56^. VeroE6-TMPRSS2 cells and VeroE6-TMPRSS2-hACE2 cells were cultured in complete DMEM in the presence of Gibco Puromycin 10 mg/mL (# A11138-03). The infectious clone SARS-CoV-2 (icSARS-CoV-2) was kindly provided to us by Dr. Vineet Menachery (UTMB)^57^. The B.1.351 variant was provided by Dr. Andy Pekosz (John Hopkins University, Baltimore, MD). Viruses were propagated in Vero-TMPRSS2 cells to generate viral stocks. Viral titers were determined by a focus-forming assay on VeroE6 cells. Viral stocks were stored at −80 °C until use.

#### Infection of mice with MA-SARS-CoV-2

B.1.351 virus was diluted in PBS to a working concentration of 1 × 10^7^ PFU/mL. Mice were anesthetized with isoflurane and infected intranasally with the virus (50 uL, 5 × 10^5^ PFU/mouse) in an ABSL-3 facility. Mice were monitored daily for weight loss. All experiments adhered to the guidelines approved by the Emory University Institutional Animal Care and Committee.

#### Quantification of infectious virus

At the indicated day post-infection, mice were euthanized via isoflurane overdose, and lung tissue was collected in Omni-Bead ruptor tubes filled with 1% FBS-HBSS. Tissue was homogenized in an Omni Bead Ruptor 24 (5.15 ms, 15 s). To perform plaque assays, 10-fold dilutions of viral supernatant in serum-free DMEM (VWR, #45000-304) were overlaid on VeroE6-TMPRSS2-hACE2 cells monolayers and adsorbed for 1 h at 37 °C. After adsorption, 0.5% immunodiffusion Agarose in 2X DMEM supplemented with 5% FBS (Atlanta Biologics) and 1× sodium bicarbonate was overlaid, and cultures were incubated for 48 h at 37 °C. Agarose plugs were removed, cells fixed with 4% PBS-buffered paraformaldehyde for 15 min at room temperature and plaques were visualized using crystal violet staining (20% methanol in ddH_2_O).

### Focus reduction neutralization titer assay

FRNT assays were adapted from Vanderheiden et al.^58^. Briefly, samples were diluted at 3-fold in eight serial dilutions using DMEM (VWR, #45000-304) in duplicates with an initial dilution of 1:10 in a total volume of 60 μl. Serially diluted samples were incubated with an equal volume of SARS-CoV-2 (100–200 foci per well) at 37 °C for 1 h in a round-bottomed 96-well culture plate. The antibody-virus mixture was then added to Vero cells and incubated at 37 °C for 1 h. Post-incubation, the antibody-virus mixture was removed and 100 µl of prewarmed 0.85% methylcellulose (Sigma–Aldrich, #M0512-250G) overlay was added to each well. Plates were incubated at 37 °C for 24 h. After 24 h, methylcellulose overlay was removed, and cells were washed three times with PBS. Cells were then fixed with 2% paraformaldehyde in PBS (Electron Microscopy Sciences) for 30 min. Following fixation, plates were washed twice with PBS and 100 µl of permeabilization buffer (0.1% BSA [VWR, #0332], Saponin [Sigma, 47036-250G-F] in PBS), was added to the fixed Vero cells for 20 min. Cells were incubated with an anti-SARS-CoV spike primary antibody directly conjugated to biotin (CR3022-biotin) for 1 h at room temperature. Next, the cells were washed three times in PBS, and avidin-HRP was added for 1 h at room temperature followed by three washes in PBS. Foci were visualized using TrueBlue HRP substrate (KPL, # 5510–0050) and imaged on an ELISPOT reader (CTL). Detailed protocol for FRNT assays can be found in Vanderheiden et al.^58^

### Quantification and statistical analysis for FRNT assay

Antibody neutralization was quantified by counting the number of foci for each sample using the Viridot program^59^. The neutralization titers were calculated as follows: 1—(ratio of the mean number of foci in the presence of sera and foci at the highest dilution of the respective sera sample). Each specimen was tested in duplicate. The FRNT-50 titers were interpolated using a 4-parameter nonlinear regression in GraphPad Prism 8.4.3. Samples that do not neutralize at the limit of detection at 50% are plotted at 10 and was used for geometric mean calculations.

### Evaluation of T cell responses

12–14 week old C57BL/6 mice were immunized with the antigen-adjuvant formulations mentioned above. On days 7 and 33 post-boost, iliac lymph nodes and lungs were harvested, processed, and a single-cell suspension was made. Lung and LN cells were plated in a 96-well round-bottom plate at a density of 2.5 × 10^5^–1 × 10^6^ cells/mL, in a 200 uL final volume with RPMI-1640 complete media containing the SARS-CoV-2 RBD overlapping peptide pool (Genscript). For unstimulated controls, media was added. Cells were cultured for 2 h, after which Brefeldin A was added, and cells were left in culture for 8 more hours. Following the stimulation, cells were stained intracellularly with an extracellular antibody cocktail containing anti-CD3 (BV785; BioLegend #100355, 1:50 dilution), anti-CD4 (BV650; BioLegend # 100555; 1:200 dilution), anti-CD8 (BV711, BD #563046; 1:200 dilution). Cells were fixed and permeabilized with BD Cytofix/Cytoperm, then stained intracellularly with a cocktail of antibodies containing anti-IFNγ (APC; BioLegend #505810; 1:100 dilution), anti-TNFα (FITC; BioLegend #506304, 1:100 dilution), anti-IL-2 (PE; BioLegend #503808, 1:100 dilution), anti-IL-4 (PerCPCy5.5; BioLegend #504124, 1:100 dilution). Cells were analyzed with an LSRII.2 analyzer at the Stanford Shared FACS Facility.

### ELISPOT assay for bone marrow plasma cells

Millipore MultiScreen 96-well plates with Immobilon-P membrane (Cat: MAIPS4510) were coated with 5 μg/ml SARS-CoV-2 spike RBD protein (Sinobiological, Cat:40592-V08B). Bone marrow cells were first treated with ACK lysis buffer to deplete red blood cells. Cells were then plated at 0.25 and 0.5 million cells/well in RPMI-1640 medium supplemented with 10% FBS, penicillin/streptomycin, glutamine, and 50 μM β-mercaptoethanol. After overnight incubation at 37 °C, RBD-specific IgG secreting cells were detected by HRP labeled goat anti-mouse IgG secondary antibody(SouthernBiotech) followed by incubation with 3-Amino-9-ethylcarbazole (AEC) substrate (BD AEC Substrate Set, Cat: 551951). ELISPOT plates were counted manually with magnification glass and normalized as the number of antibody-secreting cells/one million bone marrow cells.

### Evaluation of innate cell types

Iliac lymph nodes were harvested at day 1 post-vaccination. LNs were treated with 5 mg/ml collagenase type IV (Worthington) for 20 min at 37 °C and smashed against a 100μm strainer to make a single-cell suspension. Cells were then stained with Zombie UV™ (BUV496; Biolegend # 423108, 1:250 dilution), anti-CD64 (A488; Biolegend # 139316, 1:100 dilution), anti-Ly6C (BV780; Biolegend # 128041, 1:500 dilution), anti-Ly6G (APC-Cy7; Biolegend# 127624, 1:400 dilution), anti-CD19 (BB700; BD # 566411, 1:200 dilution), anti-CD3 (BB700; BD #742175, 1:200 dilution), anti-MHCII (AF700; eBioscience #56-5321-82, 1:400 dilution), anti-CD11b (BV650; Biolegend #101239, 1:300 dilution), anti-CD11c (BV421; Biolegend #117330, 1:400 dilution), anti-CD86 (A647; Biolegend #105020, 1:300 dilution), anti-Siglec-F (PE-CF594; BD #562757, 1:400 dilution), anti-CD24 (BUV395; BD #744471, 1:200 dilution), anti-CD45 (BV610; Biolegend #103140, 1:300 dilution), anti-CD169 (PE-Cy7; Biolegend #142412, 1:200 dilution), anti-PDCA-1 (BUV563; BD #749275, 1:200 dilution), anti-CD8a (BUV805; BD #612898, 1:200 dilution), anti-CD103 (PE; eBioscience #12-1031-82, 1:100 dilution), anti-NK1.1 (BV510; Biolegend #108738, 1:200 dilution), anti-F4/80 (BUV737; BD #749283, 1:100 dilution). Cells were analyzed with the BD FACSymphony analyzer at Stanford HIMC core.

### Mesoscale assay

V-Plex Panel 11 plates and kit were purchased from Mesoscale Discovery (Cat #: K15455U-2). Day 200 serum samples were diluted (8 different dilutions measured) and plated on the V-Plex plates. Plates were handled as per MSD protocol. Sulfo-Tag mouse secondary IgG antibody was used for the detection (Cat #: D22AGQ-3). The area under curve (AUC) was calculated for each sample.

### Statistical analysis

When comparing multiple groups to one reference group, statistical significance was determined by a one-way ANOVA followed by a Dunnett’s multiple comparisons test. When pairwise comparisons were made between all of the groups, Tukey’s multiple comparisons test was used following a one-way ANOVA. Probability values of *p* < 0.05 were considered significant and denoted by *. Where indicated, * denotes *p* < 0.0332, **p < 0.0021, ***p < 0.0002, and ****p < 0.0001. The error bars in all figures indicate SEM. The central tendency used are means, except for figures showing binding or neutralizing-antibody titers, in which case geometric means are provided.

### Ethics statement

All mice were maintained under specific-pathogen-free conditions and handled according to the approved institutional animal care and use committee (IACUC) protocols of Stanford University.

### Reporting summary

Further information on research design is available in the Nature Research Reporting Summary linked to this article.

## Supplementary information

REPORTING SUMMARY

Supplementary figures with revised author list on front page

## Data Availability

The data that support the findings of this study are available from the corresponding author upon reasonable request.
