# Supplementary material for: Adjuvanting a subunit SARS-CoV-2 vaccine with clinically relevant adjuvants induces durable protection in mice
Source: NPJ Vaccines. 2022 May 23;7:55. doi: 10.1038/s41541-022-00472-2 (PMC9126867; doi:10.1038/s41541-022-00472-2)
Supplement: Supplementary file 1 — REPORTING SUMMARY [file 41541_2022_472_MOESM1_ESM.pdf]

nature portfolio

Corresponding author(s): Bali Pulendran

Last updated by author(s): Oct 14, 2021

Reporting Summary

Nature Portfolio wishes to improve the reproducibility of the work that we publish. This form provides structure for consistency and transparency in reporting. For further information on Nature Portfolio policies, see our Editorial Policies and the Editorial Policy Checklist.

Statistics

For all statistical analyses, confirm that the following items are present in the figure legend, table legend, main text, or Methods section.

n/a	Confirmed	
☐	☒	The exact sample size (*n*) for each experimental group/condition, given as a discrete number and unit of measurement
☐	☒	A statement on whether measurements were taken from distinct samples or whether the same sample was measured repeatedly
☐	☒	The statistical test(s) used AND whether they are one- or two-sided<br>*Only common tests should be described solely by name; describe more complex techniques in the Methods section.*
☒	☐	A description of all covariates tested
☐	☒	A description of any assumptions or corrections, such as tests of normality and adjustment for multiple comparisons
☐	☒	A full description of the statistical parameters including central tendency (e.g. means) or other basic estimates (e.g. regression coefficient) AND variation (e.g. standard deviation) or associated estimates of uncertainty (e.g. confidence intervals)
☐	☒	For null hypothesis testing, the test statistic (e.g. *F*, *t*, *r*) with confidence intervals, effect sizes, degrees of freedom and *P* value noted<br>*Give P values as exact values whenever suitable.*
☒	☐	For Bayesian analysis, information on the choice of priors and Markov chain Monte Carlo settings
☒	☐	For hierarchical and complex designs, identification of the appropriate level for tests and full reporting of outcomes
☒	☐	Estimates of effect sizes (e.g. Cohen's *d*, Pearson's *r*), indicating how they were calculated

Our web collection on statistics for biologists contains articles on many of the points above.

Software and code

Policy information about availability of computer code

Data collection	BD FACSDiva software version 8.0.1 was used for collecting the flow cytometry data on the LSRII.2 machine.
Data analysis	FlowJo software v.10.0 (Treestar Inc); GraphPad Prism version 9.0.1; Adobe Illustrator 25.0. All statistical analysis are two-sided.

For manuscripts utilizing custom algorithms or software that are central to the research but not yet described in published literature, software must be made available to editors and reviewers. We strongly encourage code deposition in a community repository (e.g. GitHub). See the Nature Portfolio guidelines for submitting code & software for further information.

Data

Policy information about availability of data

All manuscripts must include a data availability statement. This statement should provide the following information, where applicable:

- Accession codes, unique identifiers, or web links for publicly available datasets
- A description of any restrictions on data availability
- For clinical datasets or third party data, please ensure that the statement adheres to our policy

The data that support the findings of this study are available from the corresponding author upon reasonable request.

Field-specific reporting

Please select the one below that is the best fit for your research. If you are not sure, read the appropriate sections before making your selection.

☒ Life sciences ☐ Behavioural & social sciences ☐ Ecological, evolutionary & environmental sciences

For a reference copy of the document with all sections, see nature.com/documents/nr-reporting-summary-flat.pdf

Life sciences study design

All studies must disclose on these points even when the disclosure is negative.

Sample size	No statistical test was used to determine the number of samples. Sample sizes were determined as appropriate to evaluate detection of large vaccine effects based on several studies done by us and others.
Data exclusions	No data were excluded from any of the analysis.
Replication	All the assays were performed once in multiple biological replicates and all the biological replicates are presented.
Randomization	The study involved the same batch of mice (C57BL/6) that were obtained from Jackson laboratories. Mice were allocated to cages with 3-4 mice per cage, and each cage was assigned to an immunization group.
Blinding	All the experiments were conducted in an unblinded way since the investigators were involved in overall conduct of the study.

Reporting for specific materials, systems and methods

We require information from authors about some types of materials, experimental systems and methods used in many studies. Here, indicate whether each material, system or method listed is relevant to your study. If you are not sure if a list item applies to your research, read the appropriate section before selecting a response.

Materials & experimental systems

n/a	Involved in the study	
☐	☒	Antibodies
☒	☐	Eukaryotic cell lines
☒	☐	Palaeontology and archaeology
☐	☒	Animals and other organisms
☒	☐	Human research participants
☒	☐	Clinical data
☒	☐	Dual use research of concern

Methods

n/a	Involved in the study	
☒	☐	ChIP-seq
☐	☒	Flow cytometry
☒	☐	MRI-based neuroimaging

Antibodies

Antibodies used

Antibody, Fluorophore, Vendor, Cat #, Clone, Lot #

anti-Ly6C, BV780, Biolegend, # 128041, HK1.4, B311364
anti-Ly6G, APC-Cy7, Biolegend, # 127624, 1A8, B294666
anti-CD19, BB700, BD, # 566411, 1D3, 0079006
anti-CD3, BB700, BD, #742175, 17A2, 0202623
anti-MHCII, AF700, eBioscience, #56-5321-82, M5/114.15.2, 2210930
anti-CD11b, BV650, Biolegend, #101239, M1/70, B335585
anti-CD11c, BV421, Biolegend, #117330, N418, B294539
anti-CD86, A647, Biolegend, #105020, GL-1, B245405
anti-Siglec-F, PE-CF594, BD, #562757, E50-2440, 0064626
anti-CD24, BUV395, BD, #744471, M1/69, 1102765
anti-CD45, BV605, Biolegend, #103140, 30-F11, B313899
anti-CD169, PE-Cy7, Biolegend, #142412, 3D6.112, B264616
anti-PDCA-1, BUV563, BD, #749275, 927, 1271522
anti-CD8a, BUV805, BD, #612898, 53-6.7, 0328533
anti-CD103, PE, eBioscience, #12-1031-82, 2E7, 2054351
anti-NK1.1, BV510, Biolegend, #108738, PK136, B326849
anti-F4/80, BUV737, BD, #749283, T45-2342, 1168211
anti-CD64, A488, Biolegend, # 139316, X54-5/7.1
anti CD3, BV785, Biolegend, #100355, 145-2C11, B303965
anti-CD4, BV650, Biolegend, #100555, RM4-5, B250896
anti-CD8, BV711, BD, #563046, 53-6.7, 0239184

anti-CD69, PECy7, Biolegend, # 104511, H1.2F3, B291440
anti-CD44, BV421, Biolegend, #103040, IM7, B306124
anti-CD45, BV605, Biolegend, #103139, 30-F11, B301866
anti-IFNy, APC, Biolegend, #505810, XMG1.2, B290393
anti-TNFa, FITC, Biolegend, #506304, MP6-XT22, B271488
anti-IL2, AF700, Biolegend, #503818, JES6-5H4, B335226
anti-IL4, PerCPCy5.5, Biolegend, #504124, 11B11, B301560
anti-IL21, PE, eBioscience, #12-7211-82, mhalx21, 2285021

Validation

All antibodies used were evaluated by the manufacturers as provided in their websites.

Animals and other organisms

Policy information about studies involving animals; ARRIVE guidelines recommended for reporting animal research

Laboratory animals

8-12 week-old female C57BL/6 mice purchased from Jackson Laboratories were used for all experiments.

Wild animals

No wild animals were used in this study.

Field-collected samples

No field-collected samples were used in this study.

Ethics oversight

All mice were maintained under specific-pathogen free conditions and handled according to the approved institutional animal care and use committee (IACUC) protocols of Stanford University. (APLAC protocol # 32681)

Note that full information on the approval of the study protocol must also be provided in the manuscript.

Flow Cytometry

Plots

Confirm that:

☒ The axis labels state the marker and fluorochrome used (e.g. CD4-FITC).

☒ The axis scales are clearly visible. Include numbers along axes only for bottom left plot of group (a 'group' is an analysis of identical markers).

☒ All plots are contour plots with outliers or pseudocolor plots.

☒ A numerical value for number of cells or percentage (with statistics) is provided.

Methodology

Sample preparation

12-14 week old C57BL/6 mice were immunized with the antigen-adjuvant formulations mentioned above. At days 7 and 33 post-boost, iliac lymph nodes and lungs were harvested, processed, and a single-cell suspension was made. Lung and LN cells were plated in a 96-well round-bottom plate at a density of 2.5 x 105 – 1 x 106 cells/mL, in a 200 uL final volume with RPMI-1640 complete media containing the SARS-CoV-2 RBD overlapping peptide pool (Genscript), anti-CD28 and anti-CD49d. For unstimulated controls, anti-CD28 and anti-CD49d were added. Cells were cultured for 2 hours, after which Brefeldin A was added, and cells were left in culture for 8 more hours.
Following the stimulation, cells were stained intracellularly with an extracellular antibody cocktail. Cells were then fixed and permeabilized with BD Cytofix/Cytoperm, then stained with intracellular antibody cocktail.

Instrument

Cells were analyzed with an LSRII.2 analyzer at the Stanford Shared FACS Facility.

Software

BD FACSDiva software version 8.0.1 was used for collecting the flow cytometry data on the LSRII.2 machine.
FlowJo software v.10.0 (Treestar Inc) was used to analyze the flow cytometry data.

Cell population abundance

No sorting was performed in this study.

Gating strategy

Cells were selected based on FSC-A vs. SSC-A, singlets were selected using FSC-A vs. FSC-H. Live CD3 T cells were used for analysis of antigen-specific T cells. CD4 and CD8 T cells were selected as CD3+ CD4+ or CD3+ CD8+. Gating strategies for innate immune cells are provided in the manuscript.

☒ Tick this box to confirm that a figure exemplifying the gating strategy is provided in the Supplementary Information.
