# Supplementary material for: Adjuvanting a subunit SARS-CoV-2 vaccine with clinically relevant adjuvants induces durable protection in mice
Source: NPJ Vaccines. 2022 May 23;7:55. doi: 10.1038/s41541-022-00472-2 (PMC9126867; doi:10.1038/s41541-022-00472-2)
Supplement: Supplementary file 2 — Supplementary figures with revised author list on front page [file 41541_2022_472_MOESM2_ESM.pdf]

Adjuvanting a subunit SARS-CoV-2 vaccine with clinically relevant adjuvants induces durable protection in mice

Lilit Grigoryan, Audrey Lee, Alexandra C. Walls, Lilin Lai, Benjamin Franco, Prabhu S Arunachalam, Yupeng Feng, Wei Luo, Abigail Vanderheiden, Katharine Floyd, Samuel Wrenn, Deleah Pettie, Marcos C. Miranda, Elizabeth Kepl, Rashmi Ravichandran, Claire Sydeman, Natalie Brunette, Michael Murphy, Brooke Fiala, Lauren Carter, Robert L. Coffman, David Novack, Harry Kleanthous, Derek O'Hagan, Robbert van der Most, Jason S. McLellan, Mehul Suthar, David Veesler, Neil P. King, Bali Pulendran

Supplementary Figure 1

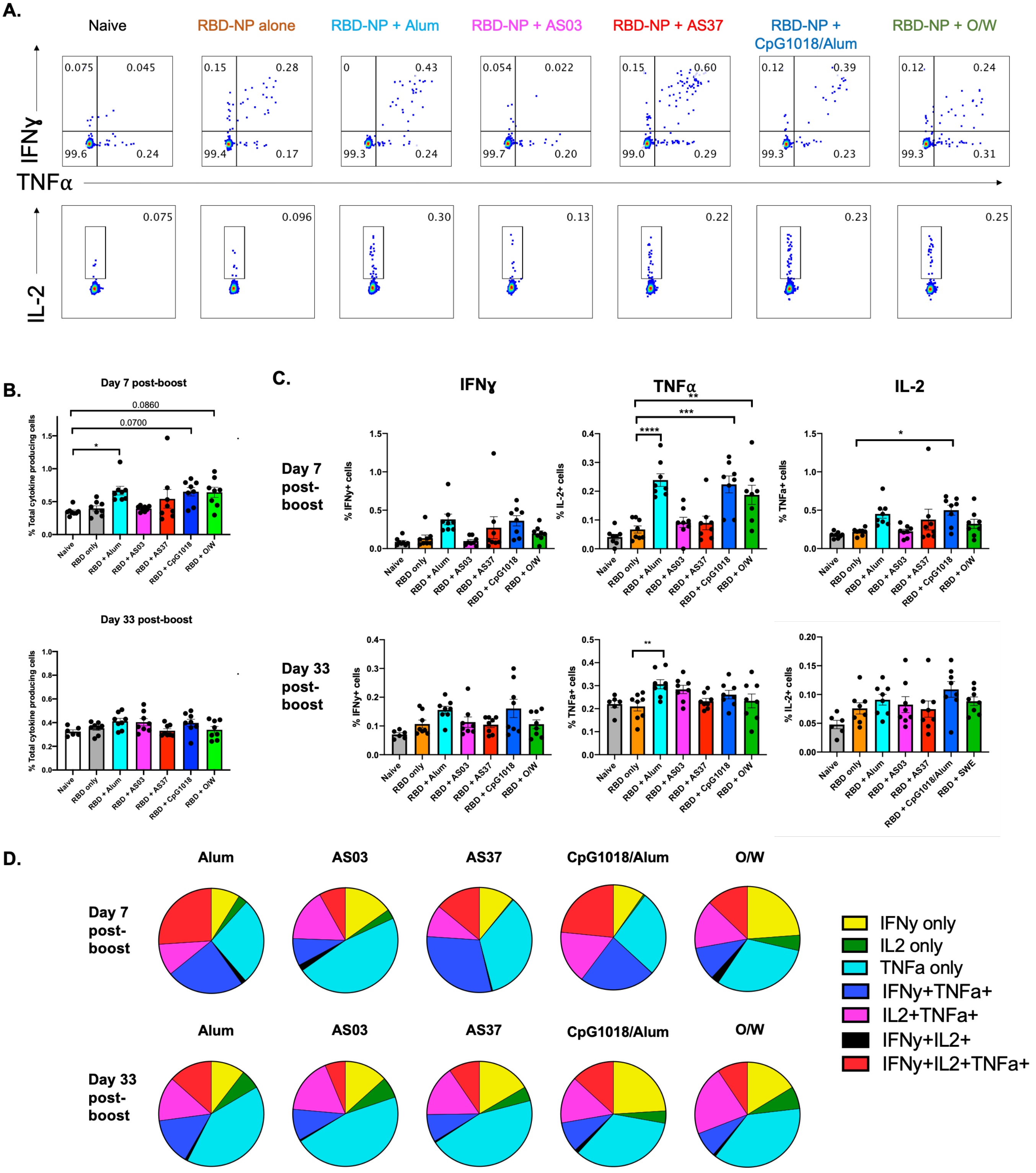

Supplementary Figure 1: Lung CD8 T cell responses. A. Representative flow cytometry plots of CD8 T cell cytokine production following stimulation with overlapping peptides from SARS-CoV-2 spike protein at day 7 post immunization. **B.** Frequency of total cytokine-producing CD8 T cells at day 7 and day 33 post immunization. **C.** Individual cytokines produced by CD8 T cells in response to overlapping peptide pool at day 7 and day 33 post immunization. **D.** Pie charts representing the average proportions of cytokine-producing cells positive for either one, two or three cytokines. (A-D) Results pooled from 2 independent experiments with n=4 mice per experiment. Error bars indicate mean±SEM in all panels.

Supplementary Figure 2

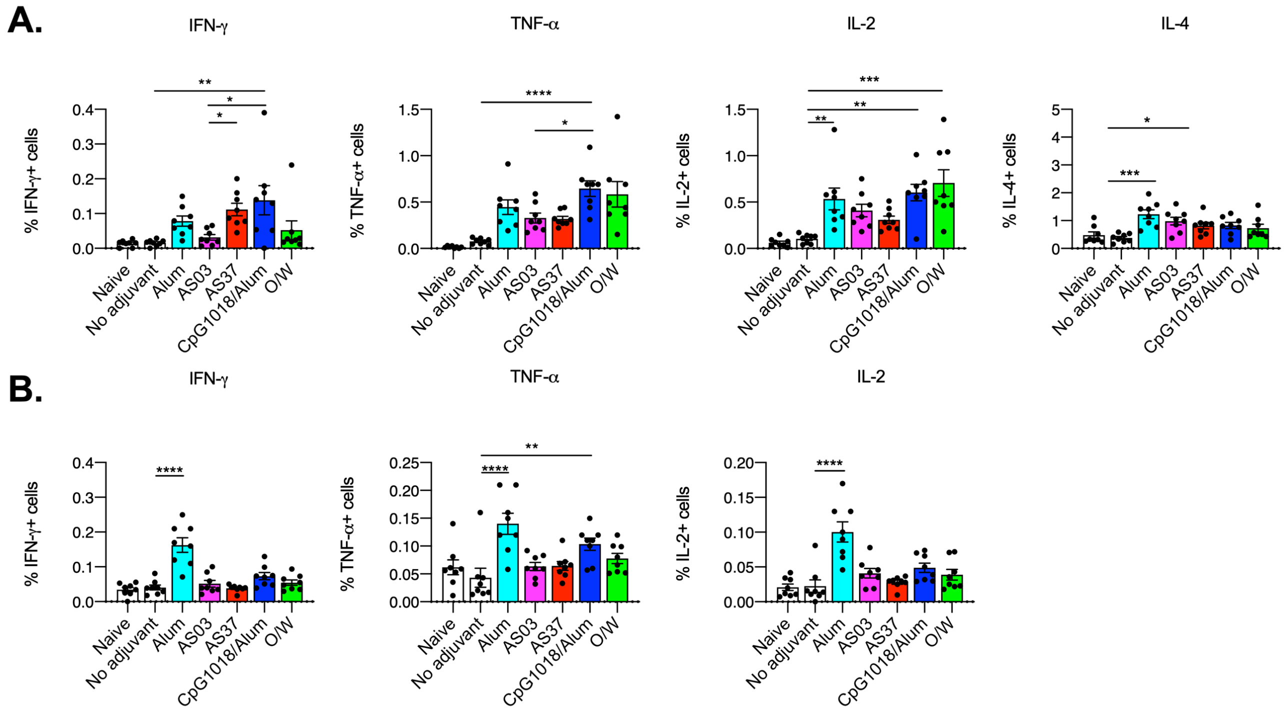

Supplementary Figure 2: T cell responses in the iliac draining lymph nodes at Day 28 (7 days psot boost). CD4 T cells shown in (A), CD8 T cells shown in (B). Results pooled from 2 independent experiments with n=4 mice per experiment. P-values: * denotes $p < 0.0332$, ** $p < 0.0021$, *** $p<0.0002$ and ****$p<0.0001$. Error bars indicate mean±SEM in all panels.

Supplementary Figure 3

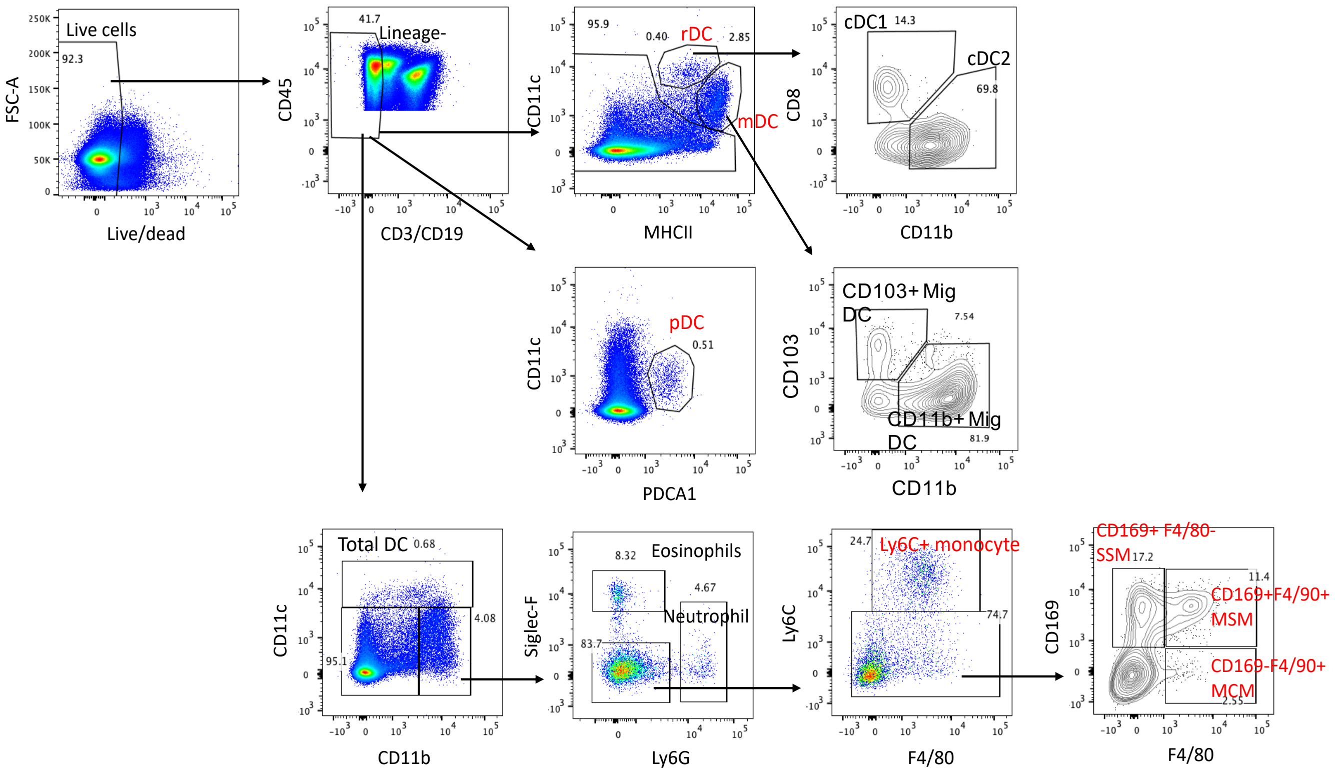

Supplementary Figure 3: Gating strategy for innate immune populations in the iliac lymph node.

Supplementary Figure 4:

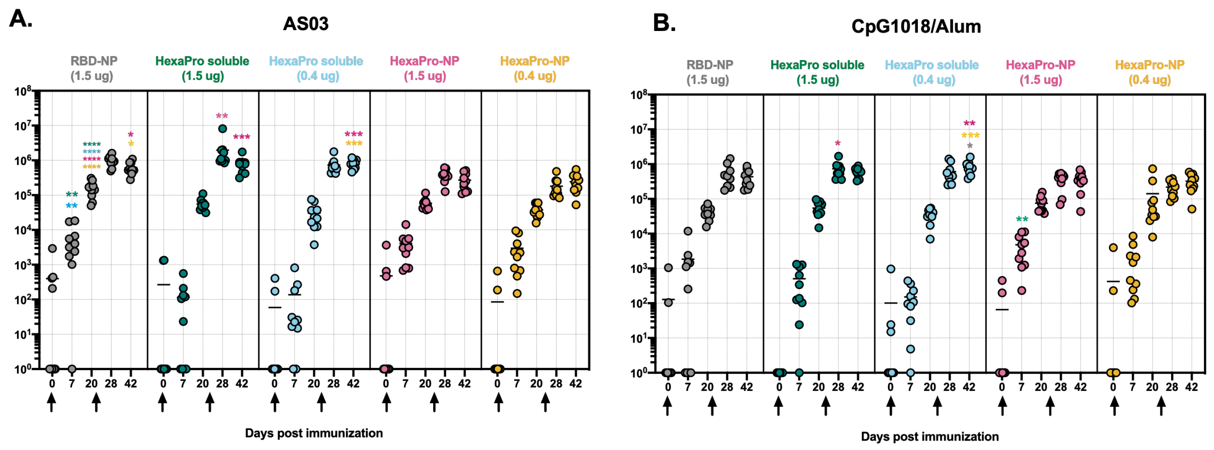

Supplementary Figure 4: Humoral Responses to immunization with other antigens at varying doses. Anti-S-2P binding antibody titers in serum of mice immunized at Days 0 and 21 with the antigen of choice combined with either (A) AS03 or (B) CpG1018/alum. Serum ELISAs were performed at timepoints on the x-axis. Error bars indicate mean±SEM in all panels.

Supplementary Figure 5:

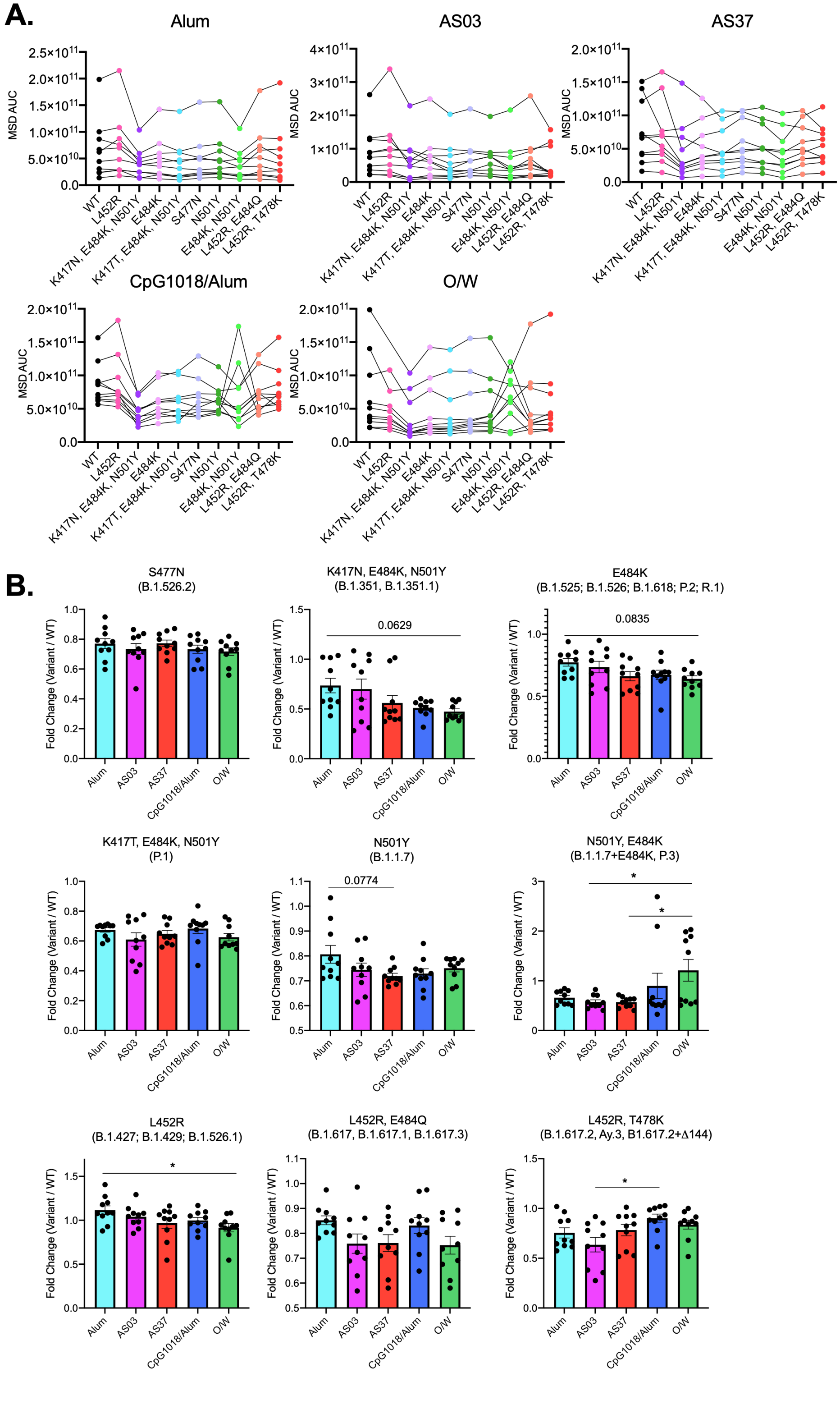

Supplementary Figure 5: Antibody responses to SARS-CoV-2 variants at Day 200 post immunization, measured by Mesoscale assay. Area under the curve (AUC) shown in **(A),** fold change of binding AUC between Variant/WT shown in **(B).** Error bars indicate mean±SEM in all panels.
